# Increased Risk of Fragility Fractures among HIV Infected Compared to Uninfected Male Veterans

**DOI:** 10.1371/journal.pone.0017217

**Published:** 2011-02-16

**Authors:** Julie A. Womack, Joseph L. Goulet, Cynthia Gibert, Cynthia Brandt, Chung Chou Chang, Barbara Gulanski, Liana Fraenkel, Kristin Mattocks, David Rimland, Maria C. Rodriguez-Barradas, Janet Tate, Michael T. Yin, Amy C. Justice, for the Veterans Aging Cohort Study Project Team

**Affiliations:** 1 VA Connecticut Healthcare System, West Haven, Connecticut, United States of America; 2 Veterans Affairs Medical Center and Department of Medicine, George Washington University, Washington, D.C., United States of America; 3 Yale Center for Medical Informatics, Yale University, New Haven, Connecticut, United States of America; 4 Section of Decision Sciences and Clinical Systems Modeling, University of Pittsburgh School of Medicine, Pittsburgh, Pennsylvania, United States of America; 5 Section of General Internal Medicine, Department of Internal Medicine, Yale University School of Medicine, New Haven, Connecticut, United States of America; 6 Section of Rheumatology, Department of Internal Medicine, Yale University School of Medicine, New Haven, Connecticut, United States of America; 7 Veterans Affairs Medical Center and Emory University School of Medicine, Atlanta, Georgia, United States of America; 8 Medical Service, Michael E. De Bakey Veterans Affairs (VA) Medical Center and Department of Medicine, Baylor College of Medicine, Houston, Texas, United States of America; 9 Division of Infectious Diseases, Department of Medicine, Columbia University Medical Center, New York, New York, United States of America; University of Cape Town, South Africa

## Abstract

**Background:**

HIV infection has been associated with an increased risk of fragility fracture. We explored whether or not this increased risk persisted in HIV infected and uninfected men when controlling for traditional fragility fracture risk factors.

**Methodology/Principal Findings:**

Cox regression models were used to assess the association of HIV infection with the risk for incident hip, vertebral, or upper arm fracture in male Veterans enrolled in the Veterans Aging Cohort Study Virtual Cohort (VACS-VC). We calculated adjusted hazard ratios comparing HIV status and controlling for demographics and other established risk factors. The sample consisted of 119,318 men, 33% of whom were HIV infected (34% aged 50 years or older at baseline, and 55% black or Hispanic). Median body mass index (BMI) was lower in HIV infected compared with uninfected men (25 vs. 28 kg/m^2^; p<0.0001). Unadjusted risk for fracture was higher among HIV infected compared with uninfected men [HR: 1.32 (95% CI: 1.20, 1.47)]. After adjusting for demographics, comorbid disease, smoking and alcohol abuse, HIV infection remained associated with an increased fracture risk [HR: 1.24 (95% CI: 1.11, 1.39)]. However, adjusting for BMI attenuated this association [HR: 1.10 (95% CI: 0.97, 1.25)]. The only HIV-specific factor associated with fragility fracture was current protease inhibitor use [HR: 1.41 (95% CI: 1.16, 1.70)].

**Conclusions/Significance:**

HIV infection is associated with fragility fracture risk. This risk is attenuated by BMI.

## Introduction

Low bone mineral density (BMD) is more common among HIV infected than uninfected individuals, potentially placing them at higher risk of fragility fracture.[1], [2], [3], [4] Studies comparing fragility fractures in HIV infected and uninfected men have been inconclusive.[5], [6], [7] In a population of 1,281 HIV infected adults on antiretroviral therapy (77% male), Collin et al. found the incidence of first fracture to be 3.3/1,000 patient-years (95% CI 2.0, 4.6). Eighty-one percent of these fractures resulted from trauma. Excessive alcohol consumption and hepatitis C coinfection were associated with higher fracture incidence while age, gender, AIDS diagnosis, baseline plasma HIV RNA, body mass index (BMI) and first protease inhibitor (PI) used were not. The incidence rate of first fractures in this study was in the same range as that reported in the general European population for the same age group. Arnsten and colleagues explored associations of HIV infection with BMD and incident fractures in HIV infected (N = 328) and at-risk men (N = 231) aged 49 years or older.[5] They reported an association between HIV infection and a decrease in BMD and between decreased BMD and increased fracture incidence. However the 38% increased fracture incidence among HIV infected men was not statistically significant, likely due to small sample size. In a larger study of HIV infected (N = 5,555) and uninfected men (N = 975,158), after controlling for age and race only, Triant *et al*. found a higher prevalence of wrist, hip, and vertebral fractures among HIV infected compared with uninfected men (3.08 per 100 persons *vs.* 1.83; *P*<0.0001).[8] This study did not include other recognized risk factors for fragility fracture.

The risk for fragility fracture is multifactoral, as has been highlighted in the World Health Organization's Fracture Risk Assessment Tool (FRAX®). These risk factors include increasing age, female gender, low BMI, previous fracture, parental history of hip fracture, current smoking, glucocorticoid use, three or more units of alcohol per day, rheumatoid arthritis, secondary osteoporosis, and decreased femoral neck BMD. Among HIV infected individuals, fragility fractures may be related to HIV-specific as well as traditional risk factors. Demographic factors, such as white race, female gender and older age increase the risk for fractures as in the general population.[5], [8] Decreased femoral BMD has also been associated with fragility fracture risk in this population.[5] The other FRAX® risk factors have not been explored in the context of HIV infection. There are also fragility fracture risk factors not included in the FRAX® but that are also associated with HIV infection. These include: chronic hepatitis B or C infection,[6], [9] chronic obstructive pulmonary disease,[10] coronary artery disease,[11] cerebrovascular disease,[12] and proton pump inhibitor use.[13] The nature of these associations likely differs by disease, thus the role that these conditions play in the relationship between HIV infection and fragility fractures may vary. As yet, the association between combined antiretroviral therapy (cART) and fragility fracture remains uncertain. The exploration of the association between cART and decreased BMD has produced inconsistent results, with some studies demonstrating an association,[3], [14] and others not.[4], [15] Exploring the contribution of individual antiretrovirals, tenofovir has been associated with an acute decrease in BMD when compared with abacavir.[16], [17] Protease inhibitors, particularly ritonavir, have been associated with decreased BMD[18], [19], [20] when compared to nonnucleoside reverse transcriptase inhibitor use.[18] An association between specific antiretrovirals and fragility fractures has yet to be determined.

## Methods

### Objectives

We explored whether the association between HIV infection and hip, vertebral, and upper arm fractures in men could be explained by traditional fragility fracture risk factors, hypothesizing that these risk factors would explain much of the association between HIV infection and fragility fractures. Among HIV infected men, we also analyzed the association between tenofovir and PI use and fragility fractures. We hypothesized that neither would be associated with an increased fracture risk.

### Sample

The Veterans Aging Cohort Study Virtual Cohort (VACS-VC) is a prospective, observational cohort of HIV infected and a 2∶1 sample of age, race, gender and site matched uninfected Veterans.[21] As described by Fultz and colleagues, the HIV infected subjects were identified by the presence of at least two outpatient or one inpatient International Classification of Diseases, Ninth Revision, Clinical Modification (ICD-9-CM) codes for HIV. Once the HIV infected subjects had been identified, each was matched to two comparators using age (five year intervals), race/ethnicity, geographic location, and gender. Comparators could not have any HIV-associated diagnostic codes. The VACS-VC represents a national sample and currently consists of 125,259 individuals (33% HIV infected) for whom demographic, pharmacy, and clinical data, including diagnostic codes, weight, height, and health factors such as smoking are available. The VACS-VC identifies all HIV infected Veterans receiving care in the Veterans Health Administration (VHA) system. These individuals enrolled for care in the VHA between 1997 and 2009. A small fraction of the HIV uninfected individuals (0.15%) seroconverted after entry into the study and were excluded from the analysis, as were those individuals to whom they were matched, 0.39% of the total sample.

### Description of Procedures or Investigations undertaken

#### Definition of Outcomes

Three fracture types were included in this study: hip, vertebral and upper arm. These were selected as they are commonly identified as resulting from a non-traumatic cause.[5], [8], [22], [23] All three types were included in a composite outcome. Unlike other authors, we excluded wrist fractures from this analysis. Wrist fractures are the third most common type of osteoporotic fracture. However they are far more common among women than men.[22] Their incidence by age differs by gender: in women, the curve is “J” shaped, indicating a likely association with osteoporosis,[24] while it is bi-modal in men, one peak between 20 and 45 and another beginning at the age of 65 years.[25] The earlier peak is thought to be more related to severe trauma than to osteoporosis.[26] As more than 70% of our sample is younger than 60 years of age, the predominant cause of wrist fractures in this group is likely trauma.[27]

Fracture outcomes were defined using the following ICD-9-CM codes: hip: 820.0X, 820.1X, 820.2X, 820.3X, 820.8, 820.9; vertebral: 805.2, 805.3, 805.4, 805.5, 805.6, 805.7; and upper arm: 812.0X, 812.1X, 812.2X, 812.3X, 812.4X, 812.5X. The use of these codes to identify fractures was validated by chart review of 400 randomly selected radiology reports. We compared the presence of ICD-9-CM codes for hip, vertebral or upper arm fractures with documentation of the fracture in radiology reports within 2 weeks before or after the ICD-9-CM fracture date. Overall agreement between the gold standard (documentation in radiology reports) and ICD-9-CM codes was 97%. Agreement beyond chance between the ICD-9-CM codes and radiologically identified fractures (kappa) was substantial (0.77, 95% CI: 0.62–0.90).[28]

#### Observation time

Observation time was calculated as the time between cohort entry (1997–2009) and date of first fracture for those with the event, or, for those who were censored, date of death or the midpoint between date of last clinic visit and the conclusion of this study (May 31, 2009), as appropriate. Individuals whose entry into the cohort was on the same date as their last follow-up were excluded from this analysis. As this analysis included time varying covariates, follow-up time was divided into six month intervals, beginning at the individual's entry into the cohort.

#### Covariates

We examined covariates identified as risk factors for fragility fracture [29], [30], [31], [32], [33], [34]: age at baseline, race/ethnicity, BMI, past or current smoking, inhaled/oral corticosteroid use, proton pump inhibitor (PPI) use, and comorbid conditions.[35] Age at baseline (time of entry into the VACS) was assessed in 10 year increments. BMI was defined as the first available BMI from no more than six months prior to enrollment. To address a possible nonlinear relationship between BMI and fracture risk, both BMI and the quadratic, BMI^2^, were included in the models. The comorbid conditions were also assessed at baseline and included congestive heart failure (CHF), pulmonary diseases, peripheral vascular disease (PVD), diabetes mellitus (DM), alcohol abuse, drug abuse, major depressive disorder, stroke, coronary artery disease (CAD), cerebrovascular disease (CVD), end stage liver disease (ESLD), cirrhosis, renal disease, chronic hepatitis B or C infection. Proton pump inhibitor use was defined as any active prescription for a PPI from study entry through time of fracture or censoring.

Among HIV infected individuals, we initially controlled for baseline CD4 count, baseline HIV-RNA levels, and use of tenofovir, PIs, and cART.[3], [36], [37], [38] Both tenofovir and PIs were included as they have been shown to be associated with decreased BMD.[39] CD4 count was categorized into 100 cell/mm^3^ increments, and HIV-RNA was log10 transformed.

Corticosteroid use, tenofovir use, PI use, and cART use were evaluated both as time-varying covariates and as cumulative exposure to drug. To accomplish this, the data were organized as multiple records per patient, each record being defined as a six month interval, as described above. In each record, two variables were created. The first noted whether or not the individual was on the drug during the time interval (yes/no), and the second recorded the cumulative time that the individual had been prescribed the medication.

### Ethics

The development of the VACS was approved by the Institutional Review Boards of the VHA Connecticut Healthcare System and Yale University School of Medicine, has been granted a waiver of informed consent, and is HIPAA compliant.[21]

### Statistical methods

Univariate statistics were used to describe the population. Chi-square and t-test were used to compare differences by HIV status.

The percent of missing data in our study was low, ranging from less than 1% to 11%. We used multiple imputation (MI)[40] via the SAS Procedure MI. The imputation model included hip, vertebral, and upper arm fractures as well as the composite outcome, the time variables for all three fracture types, and all covariates. Analyses of the imputed data sets were combined using Rubin's rule[41] as implemented in the SAS Procedure MIANALYZE. For additional information on the imputation process, please see Appendix S1.

We estimated unadjusted fracture incidence, defined as the number of hip, vertebral or upper arm fractures per 1,000 person-years. Using Cox regression models, we then adjusted for covariates previously identified as risk factors for fragility fracture. As BMI may mediate the relationship between HIV infection and fragility fracture, we first excluded it from these analyses. We then entered BMI into the models to evaluate whether it attenuated the effect of HIV infection.

Initially we explored the effect of covariates on fragility fracture risk in a combined model of HIV infected and uninfected men. We then explored models for HIV infected and uninfected men separately, including the same covariates as in the combined model. In the model for HIV infected men, when both CD4 count and HIV-RNA were included in the model, neither was significant. As they covaried (Pearson's R: 0.30), we entered each into the model separately. CD4 count was significantly associated with the outcome while log HIV-RNA was not. Therefore, only CD4 count was retained in the model. Similarly, because of covariance, cART use could not be included in models that also included PI and tenofovir use. As we were interested in exploring specific drugs that might be associated with increased fracture risk, we excluded cART use. In addition, we built one set of models that included variables for current use of corticosteroids, tenofovir, and PI. We then constructed different models that replaced the variables for current use with those for cumulative use. Cumulative use of any of these medications was not associated with fragility fractures, thus we present only the results from those models that included current corticosteroid, tenofovir, and PI use.

## Results

Our analysis included 119,318 men, 33% of whom were HIV infected (Table 1), who enrolled in VACS-VC between 1997 and 2009. There were 1615 first fractures: 496 hip, 322 vertebral, and 797 upper arm fractures. There were five instances of two fractures at the time of first fracture: four men had both vertebral and upper arm fractures and one man had both hip and upper arm fractures. None experienced three or more fractures at the time of first fracture.

**Table 1:** Sample Characteristics*.

	*HIV infected* *N = 40115*	*Uninfected* *N = 79203*	*p*
**HIV infected and uninfected men**			
Follow-up time (years) **	6.0±3.9	6.9±3.9	<0.0001
Age at time of fracture**	54±10.0	53±11	0.01
Black/Hispanic	55	55	NA†
Age >50 years at baseline	34	34	0.10
BMI (m/kg^2^)‡	25 (22, 28)	28 (25, 32)	<0.0001
Congestive heart failure	2	2	0.75
Pulmonary diseases	6	6	0.69
Peripheral vascular disease	1.0	1.3	<0.0001
Alcohol abuseDrug abuse	1619	1512	<0.0001<0.0001
Current smoker	61	54	<0.0001
Major depressive disorder	7	6	<0.0001
Coronary artery diseaseDiabetes	47	612	<0.0001<0.0001
Cerebrovascular disease/stroke	2	2	0.08
Liver disease	12	3	<0.0001
Renal disease	4	2	<0.0001
Corticosteroid use at baseline	5	3	<0.0001
Any proton pump inhibitor use	41	41	0.60
**HIV infected men only**			
Tenofovir use at baseline	3	NA	--
PI use at baseline	37	NA	--
On cART at any time during follow-up	75	NA	--
Duration of cART use**	2.9±2.5	NA	--
Baseline CD4 (cells/mm^3^)‡	274 (109, 468)	NA	--
Baseline HIV RNA (copies/mL)‡	10722 (500, 77516)	NA	--

*Data are from the baseline visit and are percents unless noted otherwise.

**Mean±SD.

† Groups matched by age and race.

‡ Median (IQR).

¥ Liver disease is a composite variable composed of chronic Hepatitis B or C infection, end stage liver disease, and/or cirrhosis.

At enrollment, 34% of HIV infected and uninfected men were over 50 years of age. Fifty five percent of subjects were black or Hispanic. Because of the matched sample design, differences in race or age were not significant. Median BMI was lower in HIV infected than in uninfected men (25 kg/m^2^ versus 28 kg/m^2^, p<0.0001). Mean follow-up time was shorter among HIV infected than uninfected men (6.0±3.9 versus 6.9±3.9, p<0.0001), and the mean age at fracture was one year older in HIV infected than uninfected men [54±10 versus 53±11 years, p = 0.01; median 53 (48, 61) versus 52 (46, 58)]. Peripheral vascular disease, coronary artery disease, and diabetes were less common among HIV infected than uninfected men (p<0.0001). In contrast, alcohol abuse, drug abuse, current smoking, major depressive disorder, liver disease, and renal disease were all more common among HIV infected compared to uninfected men (p<0.0001). HIV infected men were also more likely to have used corticosteroids at baseline than uninfected (p<0.0001). Among HIV infected men, median baseline CD4 count was 274 (109, 468) cells/mm^3^, and median baseline HIV-RNA was 10,722 (500, 77,516) copies/mL. Thirty-three percent had a serum HIV-RNA less than 1000 copies/ml at enrollment, 3% used tenofovir and 37% used a PI. Over the duration of follow-up, 75% used cART for a mean duration of 2.9±2.5 years.

In the combined model of HIV infected and uninfected men, the unadjusted incidence of all fragility fractures was 2.5/1,000 person-years among HIV infected and 1.9/1,000 person-years among uninfected men (p<0.0001). The unadjusted risk for fragility fracture was significantly higher among HIV infected than uninfected men [hazard ratio: 1.32 (95% CI: 1.20, 1.47)] (Table 2). Including common risk factors for fragility fracture, with the exception of BMI, attenuated this association, but it remained statistically significant [1.24 (1.11, 1.39)]. When BMI and BMI^2^ were included in the model, the effect of HIV was further attenuated [1.10 (0.97, 1.25)] and was no longer statistically significant. Greater BMI was associated with a decreased risk for fragility fracture [0.82 (0.79, 0.85)]. However, that BMI^2^ was also significantly associated with fracture risk [1.002 (1.002, 1.003)] implies that the risk for fracture is higher than expected at the extremes of BMI.

**Table 2:** Comparative models for any fragility fracture (hip, vertebral, or humeral) in male Veterans*.

	Model with HIV only**	Model with everything but BMI**	Full Model**	Restricted to HIV+**	Restricted to HIV-**
HIV	1.32 (1.20, 1.47)	1.24 (1.11, 1.39)	1.10 (0.97, 1.25)	--	--
Age (10 year increments)	--	1.33 (1.26, 1.41)	1.32 (1.25, 1.40)	1.52 (1.39, 1.66)	1.32 (1.25, 1.40)
White race	--	1.74 (1.56, 1.94)	1.80 (1.60, 2.03)	1.85 (1.52, 2.25)	1.80 (1.60, 2.03)
Alcohol abuse	--	1.81 (1.53, 2.15)	1.80 (1.50, 2.17)	1.50 (1.12, 2.02)	1.80 (1.50, 2.17)
Liver disease†	--	1.33 (1.08, 1.63)	1.38 (1.10, 1.73)	1.39 (1.03, 1.87)	1.38 (1.10, 1.73)
Current corticosteroid use	--	1.57 (1.28, 1.92)	1.45 (1.21, 1.74)	1.41 (1.06, 1.88)	1.45 (1.21, 1.74)
Smoker	--	1.44 (1.25, 1.66)	1.21 (1.04, 1.42)	1.30 (1.00, 1.67)	1.21 (1.04, 1.42)
Any proton pump inhibitor use	--	1.64 (1.47, 1.84)	1.70 (1.51, 1.92)	1.55 (1.28, 1.89)	1.70 (1.51, 1.92)
BMI	--	--	0.82 (0.79, 0.85)	0.87 (0.77, 0.99)	0.82 (0.79, 0.85)
BMI^2^	--	--	1.002 (1.002, 1.003)	1.002 (1.000, 1.005)	1.003 (1.002, 1.003)
CD4/100 cells/mm^3^	--	--	--	1.01 (0.98, 1.05)	--
Current TDF use‡	--	--	--	1.29 (0.99, 1.70)	--
Current PI use¥	--	--	--	1.41 (1.16, 1.70)	--

*Significant variables included in table. Other variables included in the model but not statistically significantly related to outcome: Pulmonary disease, peripheral vascular disease, major depressive disorder, coronary artery disease and/or diabetes mellitus, congestive heart failure, renal insufficiency, CD4 (per 100 cells/mm^3^), and tenofovir use. HIV-RNA was excluded from the model as it covaried with CD4 count (Pearson's R: 0.30) and only CD4 count was significantly associated with fragility fractures.

**Hazard ratio (95% confidence interval).

† Includes end stage liver disease, cirrhosis, chronic hepatitis B or C infection.

‡ TDF: tenofovir.

¥ PI: protease inhibitor.

When comparing adjusted models of fragility fracture risk in HIV infected and uninfected men, relationships between the covariates and fragility fracture were similar. As expected, older age, white race, alcohol abuse, liver disease, current corticosteroid use, smoking, and any proton pump inhibitor use increased the risk for fragility fracture in both models. In addition, among HIV infected men, an increase in BMI, but not BMI^2^, was protective against fractures. Among uninfected men, both were significant. Thus, among HIV infected individuals, the relationship with BMI was linear: a higher BMI was associated with a lower risk for fragility fracture. The relationship between BMI and fragility fracture among HIV uninfected men is as described above.

Among HIV-specific covariates, only current PI use was significantly associated with fragility fracture risk [1.41 (1.16, 1.70)]. Current tenofovir use [1.29 (0.99, 1.70)], and CD4 count at baseline [1.01 (0.98, 1.05)] were not significantly associated with fracture risk.

## Discussion

Men with HIV infection were at increased risk for incident fracture compared to uninfected, demographically similar men. Including established risk factors, particularly baseline BMI and BMI^2^, in the model attenuated the risk associated with HIV infection. In our cohort, in addition to BMI, other important modifiable risk factors included alcohol abuse, smoking, current corticosteroid use, liver disease, and any proton pump inhibitor use. As has been found in previous research, the association between corticosteroid use and bone fracture was present while the patient was on treatment.[42]

Among HIV infected individuals, current PI use was associated with an increased risk of fracture. This association was evident while the patient was on treatment. This result should be interpreted with caution. Mean follow up time for HIV infected male Veterans was 6.0±3.9 years, but mean time on cART was only half that time (2.9±2.5 years) and varied widely. Thus it is possible that PI use is a marker for disease severity and/or duration. For example, those with the most advanced disease may have initiated therapy with a PI earlier than those with less advanced disease. Thus it is not clear that PI use *per se* is associated with an increased risk for fracture. Neither CD4 count nor current tenofovir use was significantly associated with fracture risk. That tenofovir was not significantly related to fragility fracture risk may reflect its more recent emergence on the market that PI use. Tenofovir has only been available since 2001. Protease inhibitors, by comparison, have been in use since the inception of VACS.

There is limited prior work addressing the relationship between HIV infection and the risk of fragility fracture in men. Given different outcome measures and sample populations, comparisons among studies are difficult. Collin and colleagues explored the incidence of all fracture types among HIV infected men and women.[6] Triant and colleagues explored the prevalence of wrist, hip, and vertebral fractures in HIV infected and uninfected men and women, both separately and together.[8] Arnsten and colleagues explored the incidence of any fracture in HIV infected and at risk men 49 years of age and older.[5] Our study furthers this earlier work. The difference in fracture incidence between our study and that of Collin *et al.* is likely explained by their inclusion of all fracture types in both men and women. This difference may also explain why BMI was significant in our study where we limited the fractures under consideration, and not in that of Collin *et al.* Compared to the work of Triant and colleagues who explored only the role of age, race, and gender, we were able to control for a wide variety of risk factors, including modifiable risk factors, in our analysis. Our results were similar to those of Arnsten and colleagues. Remaining differences between our results and those of Arnsten and colleagues may result from the differences in outcomes: we looked at hip, vertebral and upper arm fractures while Arnsten and colleagues looked at all fractures. In addition, our sample size (N = 119,318) allowed us to explore more of the risk factors for fragility fracture than was possible in the previous study. In particular, our results support those of Bolland and colleagues who suggest that weight, or BMI, may mediate the relationship between HIV infection and low BMD.[43]

There is an extensive literature suggesting an association between decreased BMD and HIV infection.[3], [44], [45] However fragility fractures occur as a result of both decreased bone strength and injury.[46] While an important risk factor for fractures, most individuals who experience a fragility fracture do not have a BMD in the lowest range.[47], [48], [49], [50] Thus, fragility fractures and low BMD are not the same outcome.

Clinically, our work highlights the contribution of common risk factors for fragility fracture among individuals with HIV infection. The role of specific antiretroviral medications still requires further exploration. In the meantime, helping patients to stop smoking and decrease alcohol consumption is of central importance. Oral and inhaled corticosteroids and PPIs should be used sparingly. When treatment is unavoidable, providers should help patients minimize other fragility fracture risk factors and should address prevention of proximal causes of fractures, such as falls.

### Strengths

Our study has several unique strengths. These include a large and diverse national sample, incident outcomes, and data on most of the major risk factors for fragility fracture.[51] We were able to assess most of the FRAX® risk factors as well as others, including comorbid disease, alcohol abuse, smoking, corticosteroid use, and proton pump inhibitor use. Of central importance, we were also able to control for BMI. In addition, our sample is approximately a decade older than, but otherwise closely resembles, the HIV infected male population of the US: the VACS-VC male population is 44% black and 35% white while in the US HIV infected population, 46% are black and 35% white.[27] Further, our prior work has consistently demonstrated generalizability of our findings to non-Veteran populations. In three major and independent cross cohort collaborations considering associations between timing of HIV treatment and survival, associations in VACS data were consistent with those found in other cohorts from Europe and North America.[52], [53], [54]

### Limitations

Fragility fractures are far more common among women than men. However fragility fractures among men account for 20% of all fragility fractures and 25% of the costs.[46] One-third of all hip fractures occur in men.[55] Post hip fracture, one-year mortality rates are higher in men than in women (31–38% vs 12–28%, respectively)[56], and men are almost twice as likely as women to be institutionalized after a hip fracture.[57]

We were limited by the fact that we could only access data subsequent to the date of VACS enrollment. This prevented our being able to determine whether or not individuals had had a fracture prior to enrollment. In addition, as in most cohort studies, we had no information on parental history of hip fracture. We have no reason to believe that parental history of hip fracture differs by HIV status. In addition, we also used ICD-9-CM codes to identify fractures, and despite our attempts to clearly identify the fractures in our study as fragility fractures, we acknowledge that some fractures may have resulted from trauma, thus our results may overestimate fracture incidence in all subjects. Nonetheless, our relative comparisons are robust.

### Future directions

The utility of including HIV infection as a risk factor to existing fracture risk algorithms, such as the FRAX®, requires further investigation. As FRAX® is only valid in people over the age of 50 years, it cannot be utilized as a risk prognosticator in the majority of HIV infected patients, thus alternatives to the FRAX® should be explored. Finally, proximal causes of fragility fractures in the population require further exploration. As noted earlier, a combination of decreased BMD and injury cause fragility fractures. While the role of decreased BMD has been explored, that of injury, particularly falls, remains unexplored and requires elucidation.

## Supporting Information

Appendix S1 (DOC)
