# Supplementary material for: Increased Risk of Fragility Fractures among HIV Infected Compared to Uninfected Male Veterans
Source: PLoS One. 2011 Feb 16;6(2):e17217. doi: 10.1371/journal.pone.0017217 (PMC3040233; doi:10.1371/journal.pone.0017217)
Supplement: Appendix S1 — (DOC) [file pone.0017217.s001.doc]

**Appendix S1**

Multiple imputation

The percent of missing data in our study ranged from less than 1% to 11%. By using multiple imputation, we were able to include all observations in our analysis. Imputation of the outcomes using the predictors under study minimizes bias in the relationship between predictors and outcomes. [40,58]

Multiple imputation requires the assumption of data missing at random. We reduced the risk of bias from data not missing at random by including all variables of interest in the imputation models. [59] Ten imputed data sets were created using all the variables considered in these analyses. The following variables were used to impute the missing data: HIV serostatus, the outcome of interest (hip, vertebral or the composite fracture variable), race, dummy variables for age (50-59 years old, 60-69 years old, 70-79 years old, and 80 years of age or older), interaction terms between the age dummy variables and HIV serostatus, osteonecrosis congestive heart failure, pulmonary disease, peripheral vascular disease, alcohol abuse, drug abuse, major depressive disorder, coronary artery disease, diabetes, CVD, stroke, end stage liver disease, renal insufficiency, hepatitis B or C, and BMI. In models for HIV infected individuals only, CD4 and viral load were also included. Analyses of the imputed data sets were combined using Rubin’s rule,[41] as implemented in the SAS procedure MIANALYZE.
